# Sand lizard (*Lacerta agilis*) phenology in a warming world

**DOI:** 10.1186/s12862-015-0476-0

**Published:** 2015-10-08

**Authors:** Gabriella Ljungström, Erik Wapstra, Mats Olsson

**Affiliations:** Department of Biological and Environmental Sciences, University of Gothenburg, Medicinaregatan 18A, 405 30 Göteborg, Sweden; School of Biological Sciences, University of Tasmania, Private Bag 5, 7001 TAS Hobart, Australia; School of Biological Sciences, University of Sydney, Heydon-Laurence Building A08, 2006 NSW Sydney, Australia

## Abstract

**Background:**

Present-day climate change has altered the phenology (the timing of periodic life cycle events) of many plant and animal populations worldwide. Some of these changes have been adaptive, leading to an increase in population fitness, whereas others have been associated with fitness decline. Representing short-term responses to an altered weather regime, hitherto observed changes are largely explained by phenotypic plasticity. However, to track climatically induced shifts in optimal phenotype as climate change proceeds, evolutionary capacity in key limiting climate- and fitness-related traits is likely to be crucial. In order to produce realistic predictions about the effects of climate change on species and populations, a main target for conservation biologists is thus to assess the potential of natural populations to respond by these two mechanisms. In this study we use a large 15-year dataset on an ectotherm model, the Swedish sand lizard (*Lacerta agilis*), to investigate how higher spring temperature is likely to affect oviposition timing in a high latitude population, a trait strongly linked to offspring fitness and survival.

**Results:**

With an interest in both the short- and potential long-term effect of rising temperatures, we applied a random regression model, which yields estimates of population-level plasticity and among-individual variation in the average, as well as the plastic, response to temperature. Population plasticity represents capacity for short-term adjustments whereas variation among individuals in a fitness-related trait indicates an opportunity for natural selection and hence for evolutionary adaptation. The analysis revealed both population-level plasticity and individual-level variation in average laying date. In contrast, we found no evidence for variation among females in their plastic responses to spring temperature, which could demonstrate a similarity in responses amongst females, but may also be due to a lack of statistical power to detect such an effect.

**Conclusion:**

Our findings indicate that climate warming may have positive fitness effects in this lizard population through an advancement of oviposition date. This prediction is consistent over shorter and potentially also longer time scales as the analysis revealed both population-level plasticity and individual-level variation in average laying date. However, the genetic basis for this variation would have to be examined in order to predict an evolutionary response.

## Background

The global climate is changing more rapidly than ever before, having large effects on ecosystems, species and populations worldwide [1–3]. Some species have been able to track these changes, while others have been removed from their fitness peaks, decreased in numbers and in some cases gone locally or globally extinct (see [4]). One of the most frequently reported responses to recent climate change are shifts in phenology, the timing of seasonal events, where earlier emergence, migratory arrival and breeding in the spring and later migration and entrance into hibernation in autumn, have been linked to earlier spring onset and an extension of the activity season (e.g., [1–3, 5, 6]). Although observed phenological shifts are generally consistent in direction, they differ greatly in magnitude [7, 8], not only across spatial scales, but also among taxonomic groups and trophic levels [1, 2, 7, 8]. In some ecological communities this has disrupted the synchrony between predators and prey, and has led to subsequent fitness and density declines in the former ([9], reviewed by [10]). In many populations an earlier onset and prolongation of the breeding season has, however, been associated with enhanced fitness levels (e.g., [11–14]).

The vulnerability of a species to climate change is determined by a combination of intrinsic (physiological, behavioral and genetic) and extrinsic factors (ecological, regional climate change, microhabitat buffering) that dictate its sensitivity, resilience, and capacity to adapt [15]. By adapting a population can track environmentally induced shifts in optimal phenotype and hence avoid a reduction in reproductive rate. This can be achieved by dispersal to more suitable habitats, or locally by means of phenotypic plasticity or microevolution. Phenotypic plasticity, defined as the ability of a genotype to express different phenotypes across an environmental gradient [16], is a fast process that allows organisms to track rapid fluctuations in their environment. This process can therefore serve as an important means for coping with environmental change short term. However, over longer time scales the capacity for evolutionary adaptation is likely to be crucial [17], and phenotypic plasticity itself may or may not be selectively favored [18]. In order to produce realistic predictions about the fate of species and populations under ongoing climate change, a main target for conservation biologists is thus to assess the potential of natural populations to respond through these two mechanisms.

Ectotherms rely on external sources of heat to regulate their body temperature, thus ambient temperature has a strong influence on many of their basic physiological functions such as metabolism, growth, and reproduction [19], as well as on their phenology (e.g., [20, 21]), and geographic distribution [22]. This makes ecotherms particularly sensitive to changes in thermal conditions but at the same time provides them with a greater flexibility in thermal traits than their homeotherm counterparts - superficially suggesting that they would be more fitness tolerant to thermal niche shifts [23]. There is a latitudinal cline in predicted impacts of climate change on ectotherms, by which tropical ectotherms appear to be the losers. Tropical species currently live in climates very close to their optimal body temperatures (maximum performance) and their performance is thus predicted to decrease with increasing temperature [23, 24]. In contrast, at high latitudes warming could bring individuals currently at suboptimal temperatures closer to their thermal optima and thereby even enhance fitness [24]. In lizards, restricted activity time due to higher temperatures has been linked to current population declines and recent extinctions of several low latitude populations [23, 25, 26]. At high latitudes, increased activity time has, however, been predicted [27] and shown to be associated with more frequent mating and thus greater offspring viability and survival in warmer years [12, 13].

In this study we use a large 15-year individual-based data set on an ectotherm model, the Swedish sand lizard (*Lacerta agilis*; Linnaeus, 1758), to examine how year-to-year variation in spring temperature affects phenology in a high latitude population of lizards, located on the northern border of its distribution range. Specifically, we look at the effects of local temperature on oviposition date as this trait is associated with inter-annual weather variation and offspring fitness in this population [28, 29], and is connected, through its relationship with hatching date, to offspring fitness and survival in reptiles at large (e.g., [30–33]). With interest in both short- and potential long-term responses to rising temperatures (viewing temperature as a selection pressure), we test for the effect of spring temperature variation among years on the population average response, as well as for among-individual differences. Variation among individuals is a prerequisite for natural selection and hence also for an evolutionary change. As phenotypic plasticity itself may be a selective target, we explicitly test for variation among females in their average response (response in the average environment), and in their plasticity. Plasticity is examined using the reaction norm approach, which models the expression of a genotype’s different phenotypes as a function of an environmental variable [34]. Long-term individual-based data on wild populations are rare in ectotherms, hence, our study is important for understanding potential effects of climate change on species and populations within this taxon and for broader among-taxa inferences.

## Results

Our analysis included 566 records of 354 females over 15 years with a mean of 1.6 reproductive events per female. 131 females had ≥2 observations (77 females with 2 observations and 54 females with >2) and hence contributed to the estimation of variation in reaction norm slopes (see Table 1 for number of females/number of observations (reproductive events) per female). A full explanation of the data analysis is included in the methods section. In summary, we applied the following random regression mixed model for egg-laying day *LDAY* of individual *i* in response to spring temperature *TEMP*:

$$ LDA{Y}_{i, year} = u + FMAS{S}_{i, year} + EDA{Y}_{i, year} + TEM{P}_{year} + year + f\left(I{D}_{x,i},\  TEMP\right)+{e}_{i, year} $$

**Table 1:** Number of females/number of observations (reproductive events) per female

Number of reproductive events per female	Number of females
1	223
2	77
3	36
4	10
5	7
6	1

where the fixed effects covariates female mass after laying (*FMASS*) and emergence day (*EDAY*) are denoted in upper case, and the random effects year (*year*) and female identity number (*f(ID*_*x,i*_*, TEMP)*) in lower case letters, respectively. The function *f(ID*_*x,i*_*, TEMP)* allows among-individual variation around the population fixed-effect mean change in laying day over spring temperature. Fitting constant (x = 0) and linear (x = 1) functions, we tested for variation among females in their average laying date (reaction norm intercepts), and in their plastic response to temperature (reaction norm slopes). We modelled both homogeneous *e*_*i*_ and heterogeneous *e*_*i,year*_ within-individual residuals over time, and hence considered the possibility for year-specific error variances. Ignoring this possibility may erroneously yield significant among-individual variation [35, 36].

**Fig. 1:**
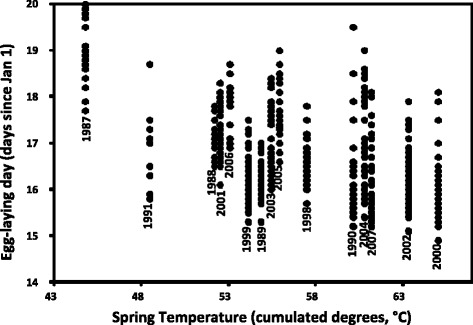
Relationship between egg-laying day and spring temperature. Descriptive plot of egg-laying day of individual sand lizard *(Lacerta agilis)* females versus spring temperature/cumulated degrees (annual cumulated sums of mean temperature over April-May in 1987–1991 and 1998–2007). The figure does not comprise all individual data points, but includes the highest and lowest number of days recorded in each year (parameter estimate −4.971 ± 1.957, *P* = 0.0244, *n* = 356)

**Fig. 2:**
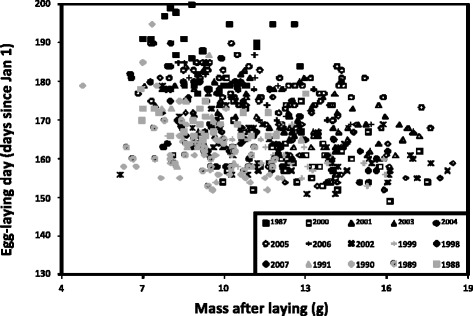
Relationship between egg-laying day and female mass. Plot of the egg-laying day (Julian days since 1 January) of individual sand lizard *(Lacerta agilis)* females versus their respective mass after laying (g). The observations are grouped into year of measurement. The figure does not comprise all individual data points, but includes the highest and lowest number of days recorded in each year (parameter estimate −1.275 ± 0.104, *P* < 0.0001, *n* = 356)

The models with a heterogeneous error structure provided a significantly better fit than those with homogeneous errors (likelihood ratio test (LRT) between homo- vs. heterogeneous models with random intercepts in Tables 3 and 4; *X*^*2*^ *= 73.5, d.f. = 14, P < 0.0001*). Hence, we only present results from models with heterogeneous residuals, but point out any differences in predictions between the two approaches. Our fifteen year data set revealed a negative relationship between laying day and spring temperature (cumulated degrees; Fig. 1) with lizards laying eggs earlier in relatively warmer years (parameter estimate −4.971 ± 1.957 (± s.e.m.), *P = 0.0244*; Table 2; Fig. 1). Confirming previous results for this population, larger females oviposited before smaller females (parameter estimate −1.275 ± 0.104 (± s.e.m.), *P < 0.0001*; Table 2; Fig. 2) and keeping female mass after laying constant, females that emerged earlier also oviposited earlier (parameter estimate 0.096 ± 0.021, *P < 0.0001*; Table 2). Independent of these effects, there was significant variation among females in average laying day (*X*^*2*^ *= 5.2, P = 0.0225*; Table 3) and among years (*X*^*2*^ *= 262.6, P < 0.0001*; Table 3).

**Table 2:** Fixed effect estimates from a mixed model of laying day

Effect	Coefficient ± s.e.m.	KR *F*	d.f.(nom)	d.f.(den)	*P*
Intercept	170.150 ± 3.722				
Cumulated degrees	−4.971 ± 1.957	6.45	1	13.3	0.0244
Emergence day	0.096 ± 0.021	21.42	1	383	<.0001
Female mass	−1.275 ± 0.104	150.53	1	445	<.0001

Parameter estimates of the most parsimonious model; random intercept model with heterogeneous among-year residuals, are given along with denominator degrees of freedom and F tests according to Kenward-Roger (KR)

**Table 3:** Solution for random effects from models fitted with *heterogeneous among-year variance*

Effect	Variance (S.E.)	−2 LogL	*χ* ^*2*^	d.f.	*P*
Null		3825.2			
Year	36.721 (15.000)	3562.6	262.6	1	<.0001
**Random intercept**	**3.633 (1.758)**	**3557.4**	**5.2**	**1**	**0.0225**
Random slope	0 (.)	3557.1	0.3	2	0.8607
Residual error	Variance (S.E.)		Residual error		Variance (S.E.)
1987	55.535 (19.263)		2001		20.434 (5.501)
1988	12.301 (4.856)		2002		25.320 (5.370)
1989	8.7973 (3.3268)		2003		18.956 (4.536)
1990	67.012 (16.763)		2004		57.974 (12.454)
1991	46.235 (20.580)		2005		18.206 (4.176)
1998	14.3431 (4.930)		2006		10.535 (4.451)
1999	16.5202 (4.585)		2007		35.037 (8.939)
2000	41.3448 (9.956)				

The most parsimonious model is denoted in bold face and the annual residual errors refer to this model. A covariance term between individual intercepts and slopes is included in the *Random slope*-model, hence d.f. = 2

Including individual plasticity in response to spring temperature ID_1_ did not significantly increase the fit of the model that included among-year heterogeneity in residuals (variance estimate 0 (.) (S.E.), *X*^*2*^ *= 0.3, P = 0.8607*; Table 3), hence, there is no statistical evidence for among-individual variation in reaction norm slopes. In contrast, the same procedure led to a significant increase in model fit when modelling homogenous residuals across years (variance estimate 10.441 (3.374) (S.E.), *X*^*2*^ *= 19.1, P < 0.0001*; Table 4), suggesting that females varied in their response to temperature. This finding shows that assuming a homogeneous error structure lead to an upward bias both in the estimate of among-individual variance and the level of significance, and hence confirms that ignoring the possibility for heterogeneity in residuals may lead to erroneous conclusions.

**Table 4:** Solution for random effects from models fitted with *homogeneous among-year variance*

Effect	Variance (S.E.)	−2 LogL	*χ* ^2^	d.f.	P
Null		3914.4			
Year	37.720 (15.338)	3635.5	278.9	1	<.0001
Random intercept	4.612 (2.416)	3630.9	4.6	1	0.0319
Random slope	10.441 (3.374)	3611.8	19.1	2	<.0001
Residual error	23.767 (2.666)				

A covariance term between individual intercepts and slopes is included in the *Random slope*-model, hence d.f. = 2

## Discussion

Phenological shifts have been observed in many species and populations in response to contemporary changes in the global climate and this is predicted to continue as climate change proceeds [1–3]. The hitherto observed consequences of these changes are variable and ultimately depend on how well organisms are able to track climatically induced shifts in optimal phenotype, thereby avoiding a loss of fitness. In this study we explored the effect of spring temperature on oviposition timing in a high latitude population of sand lizards as a first step towards understanding the potential impacts of climate change on taxon-specific phenology. Other long-term studies of wild reptile populations have reported advancements in time of reproduction in warmer years, such as earlier parturition dates of the aspis viper (*Vipera aspis*) [21], the spotted snow skink (*Niveoscincus ocellatus*) [37] and the common lizard (*Zootoca vivipara*) [38], and earlier breeding of the sleepy lizard (*Tiliqua rugosa*) [20]. Testing for population-level plasticity, we investigated how increasing spring temperature is likely to affect egg-laying date in this population in the near future. In addition, we also explored potential long-term effects by testing for among-individual variation in the average response and plasticity, a prerequisite for adaptive evolution. One other study, by Schwanz and Janzen [39], has looked at individual variation in plasticity in a phenological trait in a reptile. They investigated the potential effects of climate change on painted turtles (*Chrysemys picta*) by examining whether individual plasticity in the timing of nesting has the capacity to offset sex ratio effects due to rising temperatures.

Our analyses showed that temperatures over the reproductive period have a significant effect on oviposition date in this population; female sand lizards laid eggs earlier in relatively warmer years. This means that the females respond to fairly rapid (annual) changes in ambient thermal conditions, and hence that there is population-level plasticity in laying date. Within cohorts, earlier egg laying has previously been shown to enhance offspring fitness and survival in these lizards [29], as in reptilian offspring at large (e.g., [30–33]), which suggests that higher spring temperatures benefit components of fitness in this population, and hence that global warming may involve positive effects short term. Although contrasting with projections by Sinervo et al. [26], showing a global increase in extinction risk for lizards as a result of climate change, other studies of wild lizard populations have also presented positive predictions (see [6] for a summary). For example, Cadby et al. [37] reported earlier hatching in warmer years in two populations of the spotted snow skink (*Niveoscincus ocellatus*), which is related to increased survival in the first year of life [33]. In the common lizard (*Lacerta vivipara*), rising temperatures led to larger body size in female neonates and adults over an 18 year period, with a concomitant increase in reproductive output [30]. Specifically, in our population of *L. agilis*, higher temperature during the reproductive period has been associated with higher mating rates and number of sires per clutch, leading to increased sperm competition with positive effects on offspring survival [13]. As these findings only concern components of individual fitness, studies on the whole demography of these species might, however, yield different projections. An important selective force driving trait evolution is natural selection, which acts on among-individual differences in fitness-related traits. According to our results, individual females vary significantly in their average response to spring temperature when differences in laying date associated with body size and random variation due to annual fluctuations in environmental conditions are taken into account. Directional selection on oviposition date has previously been demonstrated for this population [29], hence, if this variation has a genetic component, an evolutionary advancement in average egg-laying date may take place as spring gets warmer. In contrast, we found no statistical evidence for among-individual variation in trait plasticity (individual reaction norm slopes). This result could reflect a true absence of variation in this trait, implying that all females respond to annual fluctuations in spring temperature in a similar way. However, another potential explanation for this apparent ‘null’ result is a lack of statistical power to detect individual variation in plasticity. Our data set contained 131 females with ≥2 observations, hence contributing to variation in reaction norm slopes, but 77 of these females were observed only twice, which limits the robustness of our slope estimation. A lack of variation in laying-date plasticity could be due to strong stabilizing selection on the shape of reaction norms as mis-timing reproduction at this high latitude location can have drastic consequences [40]. This would constrain evolution towards new reaction norm optima associated with an altered climate, however, exposure to environmental conditions outside those previously experienced by a population, under which selection has not yet had an opportunity to act, could release hidden phenotypic and genetic variation, and thereby allow for an evolutionary change [41].

Interestingly, significant variation among individuals in their plastic response was obtained when modelling within-individual residuals as homogeneous across years. This agrees with findings of several other studies that also considered both approaches [36, 37, 42–46], and confirms concerns raised that ignoring the possibility for heterogeneity in residuals may lead to erroneous conclusions (e.g., [35, 36]). Patterns in residual variances can have important implications for evolutionary trajectories of phenotypic traits as they may cause estimates of heritability to change across environments (e.g., [36, 44, 47]). Exploring how environmental conditions affect residual variances may therefore improve our understanding of how organisms respond to long-term directional climate change. Our results show that in some years, egg-laying date deviated more from the individual’s estimated reaction norm than in others, indicating heterogeneous among-year, within-individual residuals. Heterogeneity in residual variances across space or time is commonly associated with differences in environmental quality (e.g., [36, 42, 43, 47]), but we found no such associations. We speculate that timing of oviposition in this species is a function of multiple environmental factors, and hence that heterogeneous residuals reflect variation in their interactions and correlations among years [48].

Thus far, our assessment of how a warming climate is likely to affect this northern population of lizards has solely been based on potential effects on oviposition timing. Climate change is, however, likely to affect a whole suite of traits, having a combined effect on viability and, ultimately, fitness. Most studies to date investigating the vulnerability of lizards to a warming climate focus on sensitivity and adaptability of thermal physiology and behavioural thermoregulation (e.g., [23–26, 49]). However, as reproductive success is a prerequisite for the long-term persistence of a population, potential effects on reproductive traits should also be considered. Furthermore, several recent studies of conservatism of lizard thermal physiology indicate that thermal tolerance is conserved across lineages, suggesting a limited potential for local evolutionary adaptation [50–52]. In contrast, intra-specific divergence in response to variation in local thermal environment has been reported for a number of reproductive traits, including age and size at maturity [53, 54], timing of ovulation and parturition [53, 40], and sex-determination system [55]. This argues for the importance of taking reproductive traits into account when assessing population sensitivity to climate change.

In summary, our results suggest that a rise in spring temperature (such as associated with climate change) may have positive fitness effects in this northern population of sand lizards by allowing for an advancement of oviposition. Our analyses revealed both population-level plasticity and individual-level variation in average laying date, hence, this prediction is consistent over shorter and potentially also longer time scales. In contrast, evolution of laying-date plasticity towards new trait optima may be constrained by a lack of among-individual variation. To verify that an evolutionary response to selection is possible, the genetic basis for the observed individual differences should be investigated using an ‘animal model’ approach. That is, a linear mixed model using pedigree-information to estimate genetic variances and covariances of adaptive phenology [56, 57].

## Conclusion

In conclusion, this study demonstrates that higher spring temperatures benefit components of fitness in high-latitude sand lizards, and hence that global warming may involve some positive effects. This is an important result as it contrasts projections of a global increase in extinction risk for lizards as a result of climate change, thus highlighting the importance of taking reproductive traits and spatial heterogeneity in responses into account when predicting future effects of climate change on species and populations.

## Methods

### The model system and study site

The field work in this population has been described in detail elsewhere [58, 28, 12, 13] so here we only give a brief account. The sand lizard (*Lacerta agilis*) is a small ground-dwelling oviparous lizard (max 20 g) with one of the largest distribution ranges for any reptile, stretching from Sweden in the North to France in the South, and from the UK to Russia, East to West [59]. The geographic range of this species extends further north, into colder climates, than any other oviparous lizard in Europe [60] and our study population (Asketunnan), located on the Swedish West coast (N57°22, E11°59´), is on the most northern border. In this location females only lay one clutch per breeding season/year. As soon as the weather conditions permitted lizard activity, the study site was monitored every day, in 1987–1991 and 1998–2007, for lizards emerging from hibernation. Upon emergence, the lizards were identified by a permanent unique toe clip and a photographic scan, weighed (to the nearest 0.1 g), measured (snout-vent and total length to the nearest 1 mm), and had their emergence dates recorded. It is possible that some lizards were not captured on the exact day that they emerged but, as the study site was monitored every day, our estimated emergence date should be highly correlated with the actual date of emergence. When females became visibly distended with eggs they were brought into captivity and kept individually in 40 × 60 × 50 cm cages with a sand substrate and a flat rock covering a moist patch where all females laid their eggs. A 40 W spotlight was mounted at one end of the cage to provide an appropriate temperature gradient (about 18–40 °C). The cages were checked at least twice daily for new eggs, which allowed for reliable recording of oviposition date. We minimized the time between capture and oviposition (~mean of 10 days) by collecting females when they had visibly distended egg contours as removing females from their natural environment may reduce the environmental signal and keeping them in the lab could potentially reduce (or increase) within- or among-individual variation. All work carried out in this study conforms to Swedish animal welfare and conservation legal requirements; ethics permit no. 82–2011 (University of Gothenburg).

The temperature data were obtained from the Swedish Bureau of Meteorology and Hydrology (SMHI) using data from the Varberg data logger, the station closest to our field site, situated on the coast ca 50 km South Asketunnan. Although the data were not collected at the immediate field site, this logger is located in an equivalent coastal position, and thus any variation among years should reflect corresponding year-to-year variation at the Asketunnan site. We calculated annual cumulated sums (sums of daily values) of mean and maximum temperature over April-May (following [61, 62, 11]), as these months represent the reproductive period [58, 63]. Climate change is defined as a change in the statistical distribution of weather patterns, such as a change in the mean or the variability that persists for an extended period of time, ranging from decades to millions of years [64]. Therefore, our 15-year data set (including two non-consecutive time periods) can be used to examine the effects of inter-annual variation in local weather conditions, but not strictly those of changes in climate. Nonetheless, the population average response to different weather conditions among years, and individual deviations from this response, give important insights into the potential short- and long-term consequences of climate change. Therefore, the annual cumulated sums of mean and maximum temperature were used as proxies for spring climate and were submitted as predictors in the statistical analysis.

### Statistical analysis

The relative biological importance of the two temperature variables, mean and maximum temperature, in regulating timing of egg laying is not obvious. Thus, to find out which variable had the strongest association with laying date, we ran linear mixed models in Proc Mixed, SAS 9.3, with laying date in a given year as response variable and mean or maximum temperature as predictor (since including both will cause intercollinearity due to their significant correlation). Mean temperature provided the best model fit (*AIC = 3636.9* versus *AIC = 3638.2*) and was used as predictor in the subsequent analysis (hereafter called spring temperature/cumulated degrees).

Oviposition date has previously been shown to correlate with date of emergence (*EDAY*) and female mass after laying (*FMASS*) in this population (earlier emergence and greater mass leads to earlier egg-laying; [29]), hence, these variables were included as fixed-effect covariates in all our models. Emergence and laying dates were expressed in Julian days since 1 January. Year and female identity number were modelled as random effects to account for random variation among years and females, and for multiple measures per year and per female. Nonsignificant factors were removed by backward elimination if their p-value was higher than the P-to-enter value of 0.1. The effect of spring temperature on laying date was further analysed using a random regression model, following methodology similar to that of several recent studies on laying date plasticity (reviewed in [65, 11, 66]). Random regression models are commonly used in analyses investigating individual variation in phenotypic plasticity of labile traits (reviewed by [35]). The method models individual reaction norms as simple linear relationships of the phenotype on the environmental variable (E) of interest, with an intercept and a slope characterizing each reaction norm [65]. If desired, the method can be extended to nonlinear reaction norms. Individual intercepts and slopes are defined as random effects and hence the model yields estimates of the among-individual phenotypic variance (*V*_*I*_) when E = 0, and the variance in reaction norm slopes (*V*_*IxE*_), as well as the covariance of these two parameters (COV_*VIVIxE*_).

We applied the following random regression model for egg-laying day *LDAY* of individual *i* in response to spring temperature *TEMP*:

$$ LDA{Y}_{i, year} = u + FMAS{S}_{i, year} + EDA{Y}_{i, year} + TEM{P}_{year} + year + f\left(I{D}_{x,i},\  TEMP\right)+{e}_{i, year} $$

where fixed effects are denoted in upper case, and random effects in lower case letters, respectively. The function *f(ID*_*x,i*_*, TEMP)* allows among-individual variance around the population fixed-effect mean change in laying day over spring temperature. Fitting constant (x = 0) and linear (x = 1) functions we tested for variation among females in their average laying date (intercepts; *V*_*I*_), and in their plastic response to spring temperature (slopes; *V*_*IxE*_). To account for correlations between individual intercepts and slopes across environments, a covariance term COV_*VIVIxE*_ was included in the linear model. A quadratic regression function (x = 2) was also fitted but led to failure of model convergence. Hence, only linear reaction norms were considered, which agrees with the population-level response (Fig. 1).

We modelled both homogeneous *e*_*i*_ and heterogeneous *e*_*i,year*_ within-individual residuals over time, and hence considered the possibility for year-specific error variances. Failure to model heterogeneous error variances, when such are present, may statistically force the among-individual variance to vary with the environmental covariate, and may hence erroneously yield significant among-individual variation [35, 36]. Significance of model fit and random factors was assessed with likelihood ratio tests (LRT), testing the difference in the −2 log likelihood between hierarchal models against a chi-square distribution with number degrees of freedom equal to the difference in number of estimated terms [67]. The environmental variable was mean-centred (mean of zero and unit variance) such that individual intercepts represent a female’s response in the average environment, rather than when E = 0.

The number of recorded reproductive events per female in our study population varied between 1 and 6, with a mean of 1.6 (see Table 1 for number of females/number of observations (reproductive events) per female), and we included all females that had bred at least once (566 records of 354 females). Only females with at least two reproductive events contribute to variance in plasticity, hence, including all females should not have an effect on this estimate. However, by increasing the sample size this affects the precision of the estimate of variation in individual intercepts, and hence the statistical power to detect IxE (see discussion on effects of keeping versus removing individuals with few observations, and on power analysis of random regression models in [68]). In our analysis 131 females had ≥2 observations and were, hence, used for estimation of variation in reaction norm slopes. It should be noted that fitting a function with only 2 points is generally not very robust and that 77 of these females had only 2 observations (54 females >2 observations).

## Availability of supporting data

The data set supporting the results of this article is available in the Dryad Digital Repository, http://dx.doi.org/10.5061/dryad.d25s3 [69].
